# Effect of Sports and Growth on Hamstrings and Quadriceps Development in Young Female Athletes: Cross-Sectional Study

**DOI:** 10.3390/sports7070158

**Published:** 2019-06-28

**Authors:** Dai Sugimoto, Dennis R. Borg, Anna N. Brilliant, William P. Meehan, Lyle J. Micheli, Ellen T. Geminiani

**Affiliations:** 1 The Micheli Center—for Sports Injury Prevention, Waltham, MA 02453, USA; 2 Division of Sports Medicine, Department of Orthopaedics, Boston Children’s Hospital, Boston, MA 02453, USA; 3 Department of Orthopaedic Surgery, Harvard Medical School, Boston, MA 02453, USA

**Keywords:** neuromuscular development, physical maturity, sports participation, figure skaters, soccer players

## Abstract

Context: Lower extremity muscular strength may vary by different sport participation during growth process. Objective: To investigate effect of sport participation and growth by comparing strength of the hamstrings, quadriceps, and hamstrings to quadriceps strength ratio (H:Q ratio) between young female figure skaters and soccer players. Design: Cross-sectional. Settings: Laboratory affiliated with regional sports medicine center. Participants: pediatric and adolescent female athletes. Procedures: Isometric hamstrings and quadriceps strength were measured. Main Outcome Measures: Strength of the hamstrings, quadriceps, and hamstrings to quadriceps strength ratio (H:Q ratio). Statistical Analysis: Effect of sport participation and growth was analyzed through a two-way (two sports: figure skaters and soccer players; three age groups: <12 years, 13–16 years, and >17 years) analysis of covariance. Results: Hamstrings strength was significantly greater in figure skaters than soccer players. Also, hamstring strength of 13–16 years and >17 years was higher compared to <12 years. Additionally, significantly higher H:Q ratio in figure skaters compared to soccer players. Conclusions: There is effect of growth on hamstrings strength among 13–16 years and >17 years compared to <12 years. Figure skaters showed greater hamstrings strength and H:Q ratio than female soccer players.

## 1. Introduction

Figure skating is a popular winter sport, especially in countries located in northern hemisphere. In 2017, there were approximately 181,000 figure skaters who belong to the US figure skating association, and 74% of the participants are females [1]. Figure skating could be broken down into solo, pair, and synchronized events as well as by level of techniques, expression, and performance, all of which are evaluated by judges. Past observational studies indicated that figure skaters were more susceptible to overuse injuries than acute injuries [2,3,4,5,6]. An epidemiological study conducted by Dubravcic-Simunjak et al. showed that 42.8% of junior level singles female figure skaters sustained overuse injuries while only 25.0% suffered from acute injuries [7]. Of those overuse injuries, stress fractures were the most prevalent (19.8%) followed by patellar tendonitis (14.9%). Another study that focused on synchronized figure skating reported that 65.8% of female synchronized figure skaters experienced at least one overuse injury in their careers [8].

On the other hand, it has been theorized that skating athletes have a lower risk for sustaining acute injuries, such as anterior cruciate ligament (ACL) tears, when compared to other competitive female athletes [9]. Female soccer players, for instance, were known as a group of competitive athletes who have a relatively high incidence of ACL tears [10,11,12]. An epidemiological study examined athletically related injury incidence over 15 years and concluded that female soccer players demonstrated the highest ACL injury rates compared to any other female collegiate athletes such as basketball, field hockey, gymnastics, ice hockey, lacrosse, softball, and volleyball [10]. There were a few proposed mechanisms of why female athletes sustain an ACL injury more frequently than male athletes such as anatomical, hormonal, and environmental factors [13,14,15,16]. Also, several biomechanical studies identified excessive knee valgus, limited knee flexion, and abnormal ground reaction forces in a jump–landing task as major risk factors [17,18].

One factor that potentially explains the acute knee injury rate disparities between the female figure skaters and female soccer players is a neuromuscular component including the balance of muscle strength between hamstrings and quadriceps. Greater quadriceps strength relative to hamstrings strength may be contributing to hyperextension movement of knee joint, which is considered as one of the noncontact ACL injury mechanisms [19]. In contrast, the main function of hamstrings is knee flexion, which is considered as an agonist of ACL as well as a counterbalancing quadriceps function during dynamic movements. For the reasons, several studies examined strength [20] and activation [21,22,23] of the hamstrings and quadriceps in dynamic movements. One of the investigations concluded that the decreased hamstring activation and increased quadriceps activation observed among females may potentially explain increased ACL injury risk compared to males [24]. Although the muscle strength imbalance was compared between sexes in the past [25,26], studies that focused on different sports within the same sex has been scarce.

The sport of soccer requires repetitive knee extension movements to execute kicking a soccer ball, which may potentially enhance quadriceps strength greatly relative to hamstrings strength. On the other hand, figure skaters may have greater hamstring strength relative to quadriceps compared to soccer players because of the greater hip extension movements in figure skating such as stroking, both forwards and backwards. Thus, specific demands of each sport may be influencing the strength ratio between hamstrings and quadriceps. Anecdotally, recent athletes are more specialized to one sport at an earlier age than years past as well as training in a specific sport year-round without substantial off-season rest. In fact, the effect of early sports specialization and participation on sports-related injuries has been documented in several studies [27,28,29,30,31]. Additionally, female specific neuromuscular alterations such as decreased hip abductor strength, increased knee abduction moment, and increased height of center of mass during growth process were documented by longitudinal studies [32,33,34,35]. To our knowledge, however, there has been a lack of descriptive research examining potential effects of sports participation and growth on lower extremity strength within females. Understanding effect of specific demands of sports participation and process of physical maturation likely helps identifying potential contributing mechanisms of musculoskeletal injuries. Therefore, the purpose of this study was to investigate effect of sport participation and growth through comparing strength of hamstrings and quadriceps, and hamstrings to quadriceps strength ratio (H:Q ratio) between young female figure skaters and young female soccer players during growth process. Given the sport-specific movement demands of their respective sports, we hypothesized that female figure skaters would develop greater hamstrings strength and H:Q ratio (greater hamstring strength relative to quadriceps strength) relative to female soccer players as they grow older.

## 2. Methods

### 2.1. Study Design

We conducted a cross-sectional study comparing hamstrings strength, quadriceps strength, and H:Q ratio of female figure skaters and female soccer players. Because of the majority of participants were younger than 18 years, signed ascent forms were received from participants’ parents/legal guardians. Institutional review board (ethics review committee), which were formed based on the Helsinki declaration including Fortaleza Actualization in 2013, approved the protocol (IRB-P00015970) of the current study.

### 2.2. Participants

The female figure skaters were recruited in the study while they were at The Micheli Center for Sports Injury Prevention for their pre-participation physical examination. The female soccer players were also asked to participate in this study voluntarily when they visited to The Micheli Center for Sports Injury Prevention. Both female figure skaters and soccer players had their hamstrings and quadriceps strength measured at the same site. Inclusion criteria of this study were (1) female sex, (2) age range between over 8 and under 20 years old, and (3) participation of either figure skating or soccer as a main sport. Exclusion criteria were (1) surgical history of knee or hip joints, (2) surgical history of thigh, and (3) neurologic functions that does not allow maximum contraction of hamstrings and quadriceps or habitual muscle cramping in muscle strength test.

After applying the inclusion and exclusion criteria, a total of 188 eligible young female athletes (Figure skaters, N = 73; Soccer players, N = 115) participated in the current study. In the figure skaters, the total population of 80% was a combination of juvenile and intermediate level, and rest of the 20% consisted of novice figure skaters. They belonged to a local figure skating club team and routinely practiced 1–2 times per week for a total of 4–6 h each week throughout the year, except for 2–3 months in summer. The soccer players were either playing soccer in middle or high school, or belonged to a local soccer club team. They usually practiced approximately 1–2 h at a rate of 2–3 times per week and competed over the weekend year-round.

### 2.3. Instrumentations

A handheld dynamometer (Hoggan Scientific LLC, Salt Lake, UT, USA) was used to evaluate isometric strength of hamstrings and quadriceps. Four healthcare practitioners (athletic trainers) measured participants’ isometric quadriceps and hamstrings strength. The inter-rater reliability of the four raters was 85.8%.

### 2.4. Procedures

For the hamstring strength test, participants were asked to lay prone on a treatment table, with the knees flexed at 90°. A handheld dynamometer was applied to the heel, and participants were asked to move their heel toward their hip/buttock. The quadriceps strength test was performed by a seated position with 90° of knee flexion. A handheld dynamometer was placed on the distal tibia, and patients were asked to cross their arms at chest and extend their knees for approximately 2–3 s with maximum force. All the strength tests were performed with under a specific direction including verbal encouragement to promote maximum effort from participants. All participants were treated and tested in the same manner. In case if participants had a difficulty understanding and following the given instruction, several practice trials were performed. Assessments were performed two times for each muscle group on each limb. The mean values of the two measurements normalized by body mass were used for analyses.

### 2.5. Data Reduction

Incomplete or missing data of hamstrings and quadriceps strength measures were eliminated for the data analysis. Mean values of normalized right and left hamstring and quadriceps peak torque were used for comparing hamstrings strength and quadriceps strength between female figure skaters and female soccer players. The H:Q ratio was calculated by mean values of normalized right and left hamstring peak torque divided by normalized mean quadriceps peak torque values. Because of the broad age range of participants (8 to 20 years old), three different age classifications were created: <12 years, 13–16 years, and >17 years. The three age breakdowns were determined because the past studies used this classification [32,36,37]. The power analysis with α value of 0.05 and β value of 0.80 indicated each age classification needs at least 16 of sample size.

### 2.6. Statistical Analysis

Physical characteristics of the two groups including age, height, body mass, and body mass index (BMI) were compared by a *t*-test with using mean and standard deviation (SD) based on the three age classifications. Independent *t*-test was used when the variables were normally distributed. Mann–Whitney U test was applied when non-normal distribution was detected by Shapiro–Wilk test. Any differences identified in the physical characteristics between the two groups were treated as a covariate. After normalization process was completed and covariates were identified, a two-way analysis of covariance (ANCOVA)—two levels of sport (female figure skaters vs. female soccer players) and three levels of age (<12 years, 13–16 years, and >17 years)—was used to examine the effect of specific sport participation and age groups on hamstrings strength, quadriceps strength, and H:Q ratio. To examine equality of error variance, Levene’s test was used. A priori α level was selected as 0.05 to identify statistical significance, and 95% confidence interval (95%CI) was reported. Also, Bonferroni corrections were used to adjust for multiple comparisons of the ANCOVA. Partial eta squared (η^2^) was employed to express the effect size of ANCOVA. For partial eta squared, <0.010 = small, 0.011–0.059 = small to medium, 0.060–0.138 = medium to large, and >0.139 = large [38]. All analyses were performed by SPSS software (IBM, Version 21.0, Chicago, IL, USA).

## 3. Results

**Figure 1:**
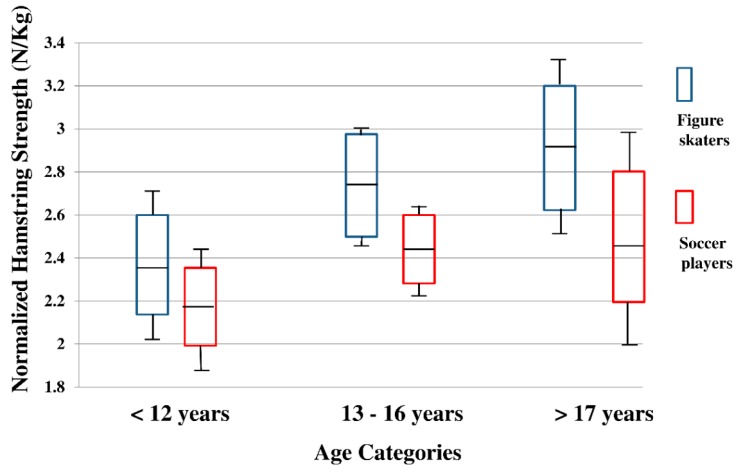
Normalized hamstring strength between female figure skaters and female soccer players in <12 years, 13–16 years, and >17 years.

**Figure 2:**
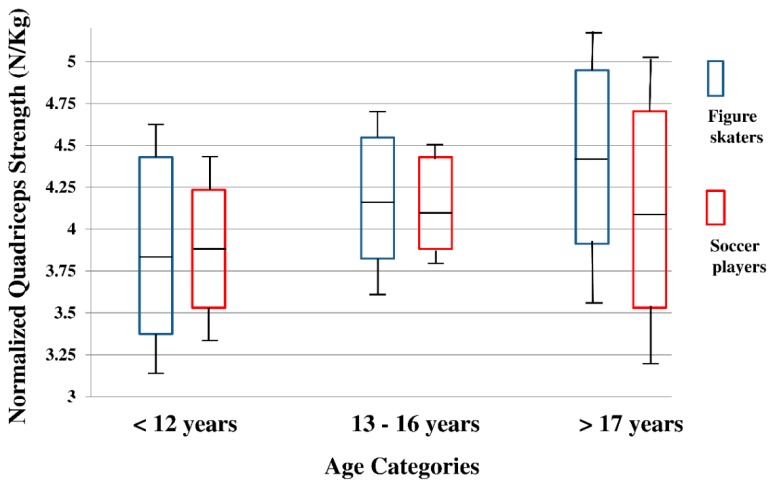
Normalized quadriceps strength between female figure skaters and female soccer players in <12 years, 13–16 years, and >17 years.

**Figure 3:**
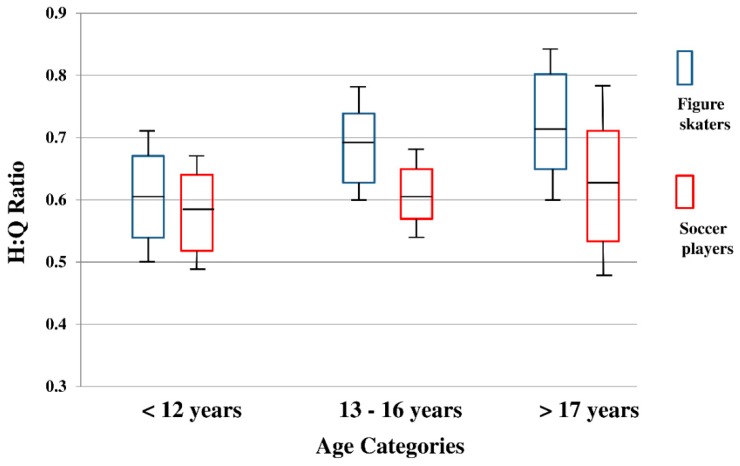
H:Q ratio between female figure skaters and female soccer players in <12 years, 13–16 years, and >17 years.

The Shapiro–Wilk test indicated that age, height, body mass, and BMI were not distributed normally so that the Mann–Whitney U test was employed. Height and body mass were different between the two cohorts (Table 1); thus, they were incorporated them in the two-way ANCOVA model. Based on the ANCOVA model, mean values with 95%CI and SD bars of hamstring strength, quadriceps strength, and H:Q ratio were illustrated (Figure 1, Figure 2 and Figure 3). Levene’s test for testing equality of error variance did not show significant values for hamstring strength (*p* = 0.297), quadriceps strength (*p* = 0.494), and H:Q ratio (*p* = 0.996). There were no significant interactions in any of the three variables. Significant main effect of sport with medium to large effect was detected in hamstrings strength (*p* = 0.001, η^2^ = 0.061, Table 2). Hamstring strength was greater in female figure skaters than female soccer players (Figure skaters, Mean: 2.67, 95%CI: (2.55, 2.79) vs. Soccer players, Mean: 2.38, 95%CI: (2.26, 2.50), Table 2). Significant main effect of age with medium to large effect was also found in hamstring strength (*p* = 0.003, η^2^ = 0.062, Table 2). Pairwise Bonferroni comparisons indicated that hamstring strength was greater in 13–16 years compared to <12 years (*p* = 0.005, <12 years, mean: 2.27, 95%CI: (2.11, 2.43) vs. 13–16 years, mean: 2.59, 95%CI: (2.48, 2.70). In addition, hamstring strength of >17 years was greater than <12 years (*p* = 0.009, <12 years, Mean: 2.27, 95%CI: (2.11, 2.43) vs. >17 years, mean: 2.71, 95%CI: (2.50, 2.92). There were no main effects of sport or age in quadriceps strength (figure skaters, mean: 4.12, 95%CI: (3.88, 4.36) vs. soccer players, mean: 4.05, 95%CI: (3.82, 4.29), Table 2). In H:Q ratio analyses, significant main effect of sport with small to medium effect was recorded (*p* = 0.022, η^2^ = 0.029, Table 2).

## 4. Discussion

The purpose of this study was to determine effect of sport participation and growth through comparing strength of hamstrings and quadriceps, and H:Q ratio between young female figure skaters and young female soccer players. We hypothesized that as female figure skaters became older, female figure skaters would develop greater hamstring strength and H:Q ratio, which demonstrate significant differences compared to female soccer players because of sport specific musculoskeletal demands in their respective disciplines. Figure skaters often repeat hip extension movements on ice while soccer players perform kicking movements, which involve knee extension. Those differences were considered and discussed to generate our hypothesis. The findings indicated that female figure skaters’ hamstring strength were significantly greater than female soccer players, and expected age effects were also detected (Table 2, Figure 1). In addition, H:Q ratio of female figure skaters was significantly higher compared to female soccer players (Table 2, Figure 3). More precisely, in the 13–16-year-old category, female figure skaters’ hamstring strength was nearly 70% of quadriceps while female soccer players’ hamstrings strength was ~60% in relation to quadriceps strength (Table 2, Figure 1).

The results may stem from the two distinctively different musculoskeletal demands of these sports. Movement patterns commonly performed in the sports of figure skating and soccer may have facilitated different strengths in hamstrings and quadriceps, which, in turn, further influenced the H:Q ratio. Physical changes led by performing different types of sports were documented [39,40]. According to a study performed by Lopezosa-Reca et al., foot posture index and quadriceps angles, commonly called “Q angles”, were significantly different between swimmers and football players [39]. Additionally, another study reported a significant bone density difference between football players and swimmers and cyclists [40]. In the current study, the lower H:Q ratio of female soccer players may be explained by the kicking motion of the soccer. Kicking motion requires knee extension movements, which may have stimulated greater quadriceps strength. The general increases of H:Q ratio in female figure skaters may stem from their stroking movements. To perform stroking movements on ice, a repetitive sequence of hip extension movements are required on ice, which might have stimulated hamstring strength development in female figure skaters over the years.

The findings of this study are unique and increase our understanding of strength development during growth process in young female athletes based on specific sports participation as well as sports specialization. A prospective, longitudinal study performed by Quatman-Yates et al. reported that a decrease of H:Q ratio from a group of female athletes who were growing from prepubertal to pubertal status. This evidence was not an agreement with the current study results. Our results showed an increase of H:Q ratio as the age becomes older. The only potential explanation is a sport effect. The athletes analyzed in the study by Quatman-Yates et al. were young female basketball players while our study group consisted of young female figure skaters and female soccer players. Main effect of sports on hamstring strength with medium to large effect was found in our study (Table 2). In addition to our study, various studies were performed to investigate effects of specific type of sports [39,40] as well as early sports specialization [27,41,42,43]. For instance, negative health outcomes of sports specialization such as increased incidences of overuse injuries in young athletes have been documented [27,30,31]. Jayanthi et al. compared a total of 1200 young athletes between sport-specialized and nonsport-specialized athletes and found that young athletes who were specialized to a sport have more likelihood to sustain serous overuse injuries such as spondylolysis, osteochondritis dissecans, and stress fracture [30]. Other studies also documented effects of sports specialization on injuries [43,44,45]; however, studies that examined effects of sport participation on physical changes were limited. The current study compared quadriceps, hamstring strength, and the ratio among young female athletes who participate in figure skating and soccer. The H:Q ratio was selected because of the clinical implications related a traumatic knee injury such as ACL injury [20,46]. The hamstrings strength as well as H:Q ratio were investigated to determine risk of knee injury such as ACL injury [20,46], and strength development of post-ACL surgery [47,48]. A matched case control study reported that teenage female athletes who demonstrated H:Q ratio of greater than 0.6 did not sustain ACL injury while those who showed H:Q ratio less than 0.6 resulted in ACL injury [20]. Similarly, another study suggested that increased torque on the ACL in side step cutting task among those who showed reduced hamstring strength [46]. Synthesizing these prior studies and the findings of this study, it may potentially be that female soccer players are predisposed to higher risk of ACL injury when compared to female figure skaters, in part, because of the lower H:Q ratio and decreased hamstring strength.

## 5. Limitations

A few limitations of this study need to be stated. First, study design of the current project was cross-sectional. It was ideal to use a prospective, longitudinal design and follow each athlete over time, which would allow us to examine biological growth and maturation more closely rather than chronological ages. However, time and financial constrains required to perform a prospective, longitudinal follow-up, which was not placed at a commencement of the current study. Therefore, we used a cross-sectional design instead. Second, approximately 85% of this cohort, both figure skaters and soccer players, were Caucasian. Although there has been no report of different lower extremity strength among specific races and ethnicities, the homogeneity of our cohort may limit generalizability. Third, inter-rater reliability of the handheld dynamometer was 85.8%. According to a study performed by Portney and Watkins [49], the inter-rater reliability of the current study was interpreted as good (0.75–0.89), but not excellent (>0.90). This may be explained by a number of raters. Several studies used two raters to examine inter-rater reliability and reported good to excellent inter-rater reliability [50,51]. The current study employed a total of four raters, which might have lowered the reliability values; however, the observed values were comparable relative to documented studies [50,51]. Fourth, the number of both female figure skaters and female soccer players in >17 years was fewer compared to the <12 years and 13–16 years age categories (Table 1). Due to the smaller number of samples, the 95%CI box plots of >17 years of the Figure 1, Figure 2 and Figure 3 were slightly larger compared to rest of the two age groups, which potentially might have influenced the outcome of this study. Moreover, there is variability (skating types, skating levels, and soccer positions) within this study population, and we did not analyze the current data based on those categories. Finally, injury data was not synthesized in this study. Sports specialization related studies often incorporate an extent of sport-related musculoskeletal injuries, especially overuse types among the physically active youth [27,30,31]. Including injury data might have shown valuable clinical findings beyond neuromuscular alteration, but it was not a scope of the current study. Instead, the current study focused on effect of early sports participation and growth on neuromuscular development in young female athletes.

## 6. Conclusions

Despite the limitations of our study, our results indicated that hamstrings strength and H:Q ratio of young female figure skaters were greater than that of young female soccer players. Also, an effect of growth was identified: hamstring strength was stronger in 13–16 years and >17 years of ages compared to <12 years. Future studies should consider incorporating injury variables for investigating effects of sports participation and physical maturation in order to generate clinically useful evidence for healthcare providers and well-being of young female athletes.

## Figures and Tables

**Table 1:** Physical characteristics of female figure skaters and female soccer players.

Demographics	Figure Skaters (N = 73)	Soccer Players (N = 115)	*p*-Value
Age (years)	13.8 ± 3.3	13.8 ± 1.9	0.937
Height (cm)	154.7 ± 15.7	161.4 ± 10.5	0.007*
Body Mass (kg)	49.7 ± 14.0	54.1 ± 10.7	0.016*
BMI	19.9 ± 3.4	20.9 ± 4.9	0.395

* indicates statistical significance. The number of participants: <12 years old figure skaters (N = 28) and soccer players (N = 30), 13–16 years old in figure skaters (N = 30) and soccer players (N = 73), and >17 years old in figure skaters (N = 15) and soccer players (N = 12)

**Table 2:** Hamstrings strength, quadriceps strength, and H:Q ratio between female figure skaters and female soccer players in <12 years, 13–16 years, and >17 years.

Strength and Ratio by Age		Figure Skaters (N = 73)	Soccer Players (N = 115)	*p*-Value	Effect Size
Hamstrings Strength (N/Kg)		2.67 (2.55, 2.79)	2.38 (2.26, 2.50)	0.001*	0.061†
Age§	<12 years	2.37 (2.15, 2.59)	2.17 (1.99, 2.36)	0.003*	0.062†
	13–16 years	2.72 (2.55, 2.90)	2.46 (2.34, 2,58)		
	>17 years	2.93 (2.66, 3.19)	2.49 (2.19, 2.80)		
Quadriceps Strength (N/Kg)		4.12 (3.88, 4,36)	4.05 (3.82, 4.29)	0.700	0.001
Age	<12 years	3.83 (3.38, 4,38)	3.90 (3.53, 4.23)	0.317	0.013‡
	13–16 years	4.17 (3.82, 4.52)	4.14 (3.90, 4.37)		
	>17 years	4.36 (3.84, 4.88)	4.13 (3.54, 4.73)		
H:Q Ratio		0.61 (0.54, 0.68)	0.58 (0.52, 0.64)	0.022*	0.029‡
Age	< 12 years	0.61 (0.54, 0.68)	0.58 (0.52, 0.64)	0.161	0.020‡
	13-16 years	0.69 (0.63, 0.74)	0.61 (0.58, 0.65)		
	> 17 years	0.72 (0.64, 0.80)	0.63 (0.53, 0.72)		

Hamstrings and quadriceps strength were normalized by weight, and units are Newton/kilogram. The values of figure skating and soccer players were expressed as mean with lower and upper points of 95% confidence interval (95% CI). * indicates statistical significance. § Bonferroni pairwise comparisons indicated a significant difference between <12 years and 13–16 years (*p* = 0.005) Also, Bonferroni pairwise analysis showed a significant difference between <12 years and >17 years (*p* = 0.009). There was no difference between 13–16 years and >17 years (*p* = 0.897) † indicates medium to large effect. ‡ indicates small to medium effect.
